# Seroprevalence and risk assessment of *Toxoplasma gondii* in Java sparrows (*Lonchura oryzivora*) in China

**DOI:** 10.1186/s12917-019-1888-7

**Published:** 2019-05-06

**Authors:** Si-Yang Huang, Yi-Min Fan, Kai Chen, Qiu-Xia Yao, Bin Yang

**Affiliations:** 1 Institute of Comparative Medicine, College of Veterinary Medicine, Yangzhou University, and Jiangsu Co-innovation Center for Prevention and Control of Important Animal Infectious Diseases and Zoonosis, and Jiangsu Key Laboratory of Zoonosis, Yangzhou, Jiangsu Province, 225009 People’s Republic of China; 2 State Key Laboratory of Veterinary Etiological Biology, Key Laboratory of Veterinary Parasitology of Gansu Province, Lanzhou Veterinary Research Institute, Chinese Academy of Agricultural Sciences, Lanzhou, Gansu Province 730046 People’s Republic of China

**Keywords:** *Toxoplasma gondii*, Java sparrows, Seroprevalence, Modified agglutination test (MAT), China

## Abstract

**Background:**

*Toxoplasma gondii*, an intracellular zoonotic parasite, infects all mammalian and birds. Understanding the prevalence of *Toxoplasma* in bird is important for evaluating the transmission of this parasite. No information about the seroprevalence of *T. gondii* in Java sparrows (*Lonchura oryzivora*) is available.

**Results:**

In this study, from 2014 to 2015, 350 serum samples from Java sparrows were collected in Beijing and Shangqiu, Henan province, and the antibodies against *T. gondii* were evaluated with MAT. The seroprevalence in Java sparrows was 34.29% (CI_95%_ 29.31–39.26). A phenomenon of seropositivity tended to increase with age were observed, but the difference is not significant. The prevalence was significant different in gender and color, which could be risk factors.

**Conclusions:**

This study firstly reported *T. gondii* seroprevalence in Java sparrows, which extended the host range of *T. gondii.* Java sparrows may pose significant transmission medium, accelerating the spread of *T. gondii* diffusion.

## Background

*Toxoplasma gondii* is an intracellular zoonotic parasite, which is capable of infecting all mammalian and humans, including birds [1]. It was stated nearly one third of the world population and about 8.0% people in China were chronically infected by this parasite [1, 2]. Infection may occur by the ingestion of vegetable or water polluted by oocysts, consumption of undercooked infected meat or by transplancental transmission [3, 4].

People consider birds are important epidemiological indicators because they can reflect the soil contamination with the oocysts of *T. gondii*. At the same time, they are important dissemination medium, which prey on small animals, especially cats. Thus*,* many studies have been carried out to investigate the prevalence of *T. gondii* among bird species around the world [5–10]*.* Due to their colorful feather and elegant posture, some species of birds has been raised as pets in China for a long history, including Java sparrows*.* Nowadays, many people like to breed Java sparrows as pets, which enhanced their economic value. Although toxoplasmosis was normally asymptomatic chronic infection in birds or chicken, a few of outbreak clinical cases were reported recently [11]. The outbreak can cause great economic loss in bird and chicken production. In captivity, a lots of different colorations were bred, including, silver/opal, cream, fawn/isabel, white, pastel and agate (which is rare within Europe captive specimens) along with the gray Java sparrow. Java sparrows mainly originated from Beijing and Henan provinces in China, where about 70% of all pet birds were raised each year.

Before toxoplasma were identified (in 1908), two reports descripted toxoplasma-like parasite in Java sparrows in 1900s [12]. There is no exact information about *T. gondii* infection in Java sparrows since that time. The aim of the present study was to survey the seroprevalence of *T. gondii* infection in Java sparrows in China.

## Methods

### The investigated sites

The examined Java sparrows in present study were collected from two cities, Beijing and Shangqiu (the main production places of pet birds) in China. Beijing is located near to the Yanshan Mountains, the average altitude is around 43.5 m, the annual precipitation is about 626 mm, and the average annual temperature is12.6 °C. Shangqiu is a city of Henan province, with the altitude ranging from 30 m to 70 m, the average annual temperature of 14.2 °C.

### Collection and preparation of samples

Samples from 350 birds, including two different color (White and Gray), were collected from markets in Beijing and Shangqiu city during 2014–2015. Blood samples were collected from the wing vein of Java sparrows and incubated at 37 °C for 1 h, kept at 4 °C for 8 h, and then centrifuged at 3500 *g* for 10 min to separate the sera. The separated sera were stored at − 20 °C for further study. Information about species, age, geographic origin, and gender were recoded from the document supplied by local veterinary practitioners.

### Serological examination

Antibodies to *T. gondii* were determined in Java sparrows by the modified agglutination test (MAT) as described previously [13]. Briefly, sera were added to the “U” bottom of 96-well microtiter plates, and diluted 2-fold starting from 1:20 to 1:320. Serum samples were diluted with serum diluting buffer (0.01 M PBS, pH = 7.2), and evaluated for anti-*Toxoplasma* IgG antibodies. The antigens were diluted with antigen diluting buffer, containing 1% bovine serum albumin (BSA), 2-mercaptoethanol and Evans blue dye solution. Birds sera with MAT titers of 1:20 or higher were considered positive for *T. gondii* infection based on a previous study, positive and negative sera of house sparrows were included as positive and negative controls, respectively, and the doubtful results were re-tested immediately [6, 14].

### Statistical analysis

A Chi-square test by SAS were used to analyze the differences in the prevalence of *T. gondii* infection Java sparrows among different variables such as region, color, gender and age, were analyzed using (Statistical Analysis System, Version 8.0). It was considered statistically difference when *P* < 0.05. The variables associated to the risk of infection were analyzed in the binary Logit model as independent variables by forward stepwise regression analysis to test the seroprevalence in the multivariable regression analysis. The effects could be included in the model when *P* < 0.05.

## Results

A total of 120 (34.29%, CI_95%_ 29.31–39.26) out of the 350 Java sparrow serum samples were positive for *T. gondii* antibodies analyzed by MAT, with antibody titers of 1:20 in 29, 1:40 in 44, 1:80 in 33, 1:160 in 8, and 1:320 in 6 samples. There was no statistically difference in the seroprevalence of *T. gondii* between Beijing (38.04%, CI_95%_30.58–45.49) and Shangqiu (31.02%, CI_95%_ 24.38–37.65, *P* = 0.16). The seroprevalence in different age groups ranged from 26.09% (CI_95%_17.11–35.06) to 45.71% (CI_95%_ 36.19–55.24) (Table 1), the seroprevalence increased with age, supporting that seropositivity is related to exposure to *T. gondii*, but the difference was not statistically significant (*P* = 0.09). There was a statistically different between female (54.63%, CI_95%_85.61–98.76) and male (25.21%, CI_95%_ 16.58–26.08) (*P* < 0.01). From the results we found that Gray Java sparrow had a higher seropositivity (47.20%, CI_95%_ 38.45–55.95) comparing to white Java sparrow (27.11%, CI_95%_ 9.27–18.28), and the prevalence was significant different (*P* < 0.05). According to the multivariable regression analysis, colors and gender could be risk factors (Table 1).

**Table 1:** *Toxoplasma gondii* prevalence in Java sparrows from Beijing and Shangqiu, China

Factor	Category	No. positive	No. tested	Prevalence (%) (CI_95%_)	*P* value
Region	Beijing	62	163	38.04 (30.58–45.49)	0.16
	Shangqiu	58	187	31.02 (24.38–37.65)	
color	White Java sparrow	61	225	27.11 (9.27–18.28)	< 0.05
	Gray Java sparrow	59	125	47.20 (38.45–55.95)	
gender	female	59	108	54.63 (85.61–98.76)	< 0.01
	male	61	242	25.21 (16.58–26.08)	
Age	3~5 month	24	92	26.09 (17.11–35.06)	0.09
	6~8 month	48	153	31.37 (24.02–38.71)	
	> 8 month	48	105	45.71 (36.19–55.24)	
Total		120	350	34.29 (29.31–39.26)	

## Discussion

Due to the special transmission of birds in *T. gondii*, the epidemic of *T. gondii* infection has been studied more and more in recently. This study, for the first time, reported seroprevalence of *T. gondii* in Java sparrows in the world. Although there were two reports descripted toxoplasma-like parasite in Java sparrows in 1900s [12], no further study was reported since that time. Here, our study confirmed that this species of bird is an intermediate host for *T. gondii* using MAT for detection of anti-*T. gondii* antibodies. MAT was developed by professor Dubey in 1987, based on detection of IgG antibodies [13], which has been extensively used for seroprevalence studies in the recent years [6, 15–18].

In this study, the prevalence of *T. gondii* in Java sparrows was 34.29%, which was higher than that in other pet birds, such as in *Carduelis spinus* (11.65%), in *Alauda gulgula* (11.39%) and in *Cocothraustes migratorlus* (5.26%) [6], in pigeons (11.86%) [19]. These examine method used in above studies was the same with our study, so the seroprevalence differences were not due to the detection method. Thus, various species and sample collected regions may contribute to the differences.

Beijing and Shangqiu were selected for screening *T. gondii* prevalence because they were the main supply areas of the pet birds. In the present study, the seroprevalence of *T. gondii* in Java sparrows is 38.04% in Beijing and 31.02% in Shangqiu. The different geographical conditions, especially climates and environment of the two regions may lead to the difference. Although the seroprevalence in Beijing was higher than in Shangqiu, the difference was not statistically significant (*P* > 0.05). Recently, a study reported that *T. gondii* prevalence of pet parrots in Beijing is 8.36%, which is much lower comparing to our result in the same area [20]. The difference might due to the different species of bird, probably, the Java sparrows is more sensitive to *T. gondii* than parrots, or other reasons (food and environment of farm) caused this difference.

In the present study, comparing to White Java sparrow, the seroprevalence of Gray Java sparrow (47.20%) was quite high, and the different was significant (*P* < 0.05). This difference probably resulted from the different color of Java sparrows, the Gray Java sparrows may be easy infected by *T. gondii* comparing to the White Java sparrows. But the detail reasons need to be further study. For the different gender, the positive rate in female was 54.63%, which is more than two times higher than the rate in male (25.21%), and the different was statistically significant (*P* < 0.01). The similar phenomenon was also observed in mice, the female mice were found to be more susceptible to acute infection [21].. So, the female Java sparrows are higher at risk of acquiring *T. gondii* infection compared to male.

Felids are essential in the life cycle of *T. gondii*, In 2013–2014, a seroprevalence and genetic characterization study was reported that 50% of cats collected from Central China (Henan, 257, Beijing, 61) were positive for T. gondii infection [22]. Our samples were also collected from these two area, the high seroprevalence of cats may cause related to the high prevalence of Java sparrows because cats play an important role in the transmission of *T. gondii*. For Java sparrows, most of the infection was happened at the early breed time. At this time, birds were usually bred in semi-free range systems, hundreds of birds were kept in a big bird house. Birds got infected by the food or water which polluted by the oocyst delivered along the cat feces. Before or after the birds were sold to the individual host as pets, the dead bodies were normally treated improperly, most of them were through to rubbish bin directly, and then eat by stray cats, completing the transmission cycle. Birds could transmit the parasite in a long distance, and this transmission route accelerated the spread of *T. gondii* diffusion. Although *T. gondii* is prevalence in stray dogs [23], the transmission effectiveness is lower comparing to cats. The population and the prevalence of toxoplasmosis in cats may affect transmission of the disease to birds. The seroprevalence of *T. gondii* in birds in different regions also may reveal the transmission of the disease in these regions. In this study, there was no significant difference between the two geographic areas surveyed. This could have resulted from the same prevalence of stray cats in these two places. Birds produced in Beijing and Henan were traded throughout China [20], which may serve as a source for *T. gondii* transmission. Therefore, the results of the present study provided basic knowledge to control the *T. gondii* transmission through bird trade route. PCR method should be included to confirm the infection as our previous reports [6, 14], while it is a pity that tissue samples of Java sparrows were not available in this study, the other detecting methods and Isolation of the parasite from infected bird were not performed.

## Conclusions

The results of the present study revealed an overall *T. gondii* seroprevalence of 34.29% in Java sparrows in China, which may pose significant transmission medium, accelerating the spread of *T. gondii* diffusion. To our knowledge, our study is the first report of *T. gondii* prevalence in Java sparrows, which also extends the host range of *T. gondii.*

## Abbreviations

BSA: Bovine serum albumin
ELISA: Enzyme-linked immunosorbent assay
IFA: Indirect immunofluorescence assay
MAT: modified agglutination test
PBS: phosphate buffered saline
